# Face validity of a dyslexia screening instrument for secondary students

**DOI:** 10.1371/journal.pone.0353781

**Published:** 2026-07-20

**Authors:** Nik Zatul-Iffah N. Mohd Nabil, Mohd Effendi Ewan Mohd Matore, Mohd Syazwan Zainal

**Affiliations:** 1 Faculty of Education, The National University of Malaysia, Bangi, Selangor, Malaysia; 2 Research Centre of Leadership and Educational Policy, Faculty of Education, The National University of Malaysia, Bangi, Selangor, Malaysia; 3 Research Centre of Education and Community Well-being, Faculty of Education, The National University of Malaysia, Bangi, Selangor, Malaysia; Minnan Normal University, CHINA

## Abstract

Dyslexia is a specific learning difficulty marked by persistent challenges in reading accuracy, fluency, and word recognition despite adequate intelligence and instruction. In secondary schools, many cases remain unidentified due to the absence of screening instruments designed for older students. Early identification is essential for timely intervention, which requires valid and reliable tools. This study evaluated the face validity of a newly developed dyslexia screening instrument for secondary students through expert and test-taker assessments. The 28-item instrument, which focused on cognitive manifestations of dyslexia, was reviewed by ten experts and ten students. Items were rated on a 4-point Likert scale assessing clarity and comprehensibility, with analyses conducted using the Item-Level Face Validity Index (I-FVI), Scale-Level Face Validity Index by Average Agreement (S-FVI/AVE), and Scale-Level Face Validity Index by Universal Agreement (S-FVI/UA). Expert evaluation demonstrated that 85.7% of items achieved full agreement (I-FVI = 1.00), while scale-level indices exceeded recommended benchmarks (S-FVI/AVE = 0.96; S-FVI/UA = 0.86). Student evaluation also yielded high scale-level agreement (S-FVI/AVE = 0.92; S-FVI/UA = 0.82). A small number of items required linguistic refinement. Consequently, the findings indicate high agreement on the instrument’s clarity, comprehensibility, and acceptability, supporting face validity as a preliminary validation step for subsequent psychometric validation.

## Introduction

Early identification of dyslexia is crucial in both educational and clinical settings [1–3], as it enables timely intervention that can significantly enhance academic outcomes and cognitive development. Research has revealed that early screening, particularly during preschool years [1,4,5], can effectively identify children at risk of dyslexia, allowing for targeted interventions that improve literacy skills and long-term educational success [6,7]. Without early detection, children with dyslexia often experience persistent reading difficulties, leading to academic underachievement and negative socio-emotional consequences [8–11]. This issue is directly linked to Sustainable Development Goal (SDG) 4: Quality Education, which emphasizes inclusive and equitable education, ensuring that all learners, regardless of learning difficulties, have access to appropriate support mechanisms [12]. Developing reliable and valid screening tools is therefore essential in achieving this global objective. Given the profound impact of early screening, the development of reliable and valid dyslexia screening tools remains a priority in educational psychology and special education [13,14].

Despite the availability of various dyslexia screening instruments, several challenges remain. Existing tools may demonstrate inconsistencies in identifying students at risk across different linguistic and cultural contexts. [15,16]. In addition, misconceptions about dyslexia among educators and practitioners may contribute to misidentification and ineffective intervention strategies. Additionally, research indicates that educators and professionals may hold misconceptions about dyslexia, potentially leading to misdiagnosis and ineffective intervention strategies. [17–19]. Recent narrative reviews of school-based dyslexia screening tools have also highlighted considerable variation in the instruments used across educational contexts, with no clear consensus on the most appropriate screening approach. The diversity of tools, as well as differences in reported sensitivity and specificity, indicates the need for contextually appropriate screening instruments tailored to specific educational populations [20].

To address this gap, the Face Validity Index (FVI) provides a structured quantitative approach for evaluating item clarity and comprehensibility through systematic expert and user appraisal [21]. Instruments with high face validity are more likely to be trusted, accepted, and effectively implemented in real-world educational and clinical settings [22]. Although the FVI has been increasingly applied in health and educational measurement studies, its application in the development of dyslexia screening instruments, especially for secondary school populations, remains limited. Most existing dyslexia screening tools focus primarily on early childhood or primary school populations, leaving a gap in instruments specifically designed for adolescents whose reading difficulties may have been previously unidentified. In addition, few studies have incorporated both expert judgement and test-taker perspectives within the face validity evaluation process, despite recommendations advocating stakeholder involvement during early stages of instrument development [23]. Therefore, this study contributes to the literature by developing and evaluating the face validity of a dyslexia screening instrument tailored for secondary school students, addressing an important gap in current screening practices.

Accordingly, this study focuses on face validity as a foundational phase of instrument development, conducted prior to subsequent construct and reliability validation. The study aims to evaluate the face validity of a newly adapted dyslexia screening instrument for secondary school students using the Face Validity Index. Item clarity, relevance, and comprehensibility were examined through structured assessments involving expert educators and secondary school students as test takers. This investigation represents the initial stage of a broader validation process, providing empirical evidence of user acceptability and linguistic suitability before advanced psychometric analyses are undertaken. By establishing foundational validity, the study contributes to the development of accessible and contextually appropriate screening tools that support early identification and inclusive educational practices aligned with SDG 4.

### Validity in instrument development

Validity is a cornerstone concept in psychometrics, referring to the extent to which an assessment tool accurately evaluates the construct it is intended to measure [24,25]. It encompasses various forms, including construct validity, content validity, criterion-related validity, and face validity, each serving a unique purpose in the evaluation of an instrument’s effectiveness. Notably, construct validity ensures that the tool accurately captures the theoretical concept under investigation, while content validity ensures that the test items adequately represent the domain of interest [26]. In educational and psychological assessments, validity is critical for establishing the reliability of data-driven decisions and interventions.

### Face validity in educational and psychological measurement

Face validity, defined as the extent to which an assessment appears relevant and understandable to non-expert users, such as educators, parents, and even students, significantly influences the tool’s acceptability and implementation [27,28]. Although face validity does not provide statistical evidence of measurement accuracy, it plays a crucial role in determining user acceptance, compliance, and real-world applicability. Instruments that lack face validity may be rejected by practitioners or misunderstood by respondents, reducing their effectiveness regardless of underlying psychometric strength [22].

In educational contexts, face validity is particularly salient for screening tools used in school settings, where teachers and students are required to engage with items directly. For students with learning difficulties such as dyslexia, linguistic clarity and cognitive accessibility are essential to prevent response bias and misinterpretation [29,30]. Consequently, face validity serves as a bridge between technical measurement design and functional usability.

Despite its importance, face validity has historically been underreported or treated informally in instrument development studies, often described qualitatively without systematic evaluation. Recent methodological literature, however, has highlighted the need for structured and transparent approaches to face validity assessment, particularly during early stages of scale development [23,31].

### Face validity index (FVI)

The Face Validity Index (FVI) provides a quantitative framework for evaluating face validity through systematic ratings of item clarity and comprehensibility by experts and intended users [21]. Unlike purely descriptive approaches, the FVI offers numerical indices at both item level (Item-Level Face Validity Index, I-FVI) and scale level (Scale-Level Face Validity Index, S-FVI), allowing researchers to make evidence-informed decisions regarding item retention, refinement, or revision.

Although no single scholar is universally recognized as the originator of the FVI, its structured application has been clearly articulated in methodological work by [21], who outlined standard procedures for rating, recoding, and interpreting face validity data. The FVI is a quantitative approach to assess the degree to which test items appear effective [32], valid [33], and relevant to stakeholders, such as educators, parents, and clinicians. Furthermore, the FVI is also referred to by various synonymous terms in the literature, including face validity score [30] and expert agreement index [34], particularly in linguistic and usability studies [35]. It involves evaluators rating each item based on clarity, relevance, and comprehensibility, with the FVI calculated as the proportion of items deemed valid by the assessors [21]. Consequently, this metric provides an empirical basis for determining whether an instrument is likely to be accepted and utilized effectively in real-world settings. In the context of dyslexia screening, a high FVI indicates that the tool is perceived as appropriate and user-friendly, which is essential for its successful implementation in educational environments.

Moreover, several studies [36–39] have employed the FVI to enhance the development of educational and psychological assessments. For instance, the Dyslexia Adult Checklist underwent a validation process where items were evaluated for clarity and relevance, leading to a refined instrument that effectively identifies dyslexia in adults [13]. Similarly, the Teacher Teaching Quality Instrument using the Six Sigma approach (T^2^Qi-6σ) was subjected to face validity assessment [40], resulting in a tool that accurately measures teaching quality and supports SDGs related to quality education. These examples underscore the importance of incorporating FVI in the validation process to ensure that assessment tools are both effective and aligned with educational objectives.

Conversely, incorporating the FVI in the development of dyslexia screening instruments is particularly crucial due to the diverse range of stakeholders involved, including teachers, parents, and policymakers. A screening tool with a high FVI is more likely to be embraced by educators and parents, facilitating early identification and intervention for children with dyslexia. This supports inclusive education efforts [12]. Thus, by utilizing the FVI, developers can create culturally sensitive and contextually appropriate tools that address the specific needs of various populations, thereby promoting educational equity and supporting the global agenda for sustainable development.

### Dyslexia screening instrument

Dyslexia screening instruments are brief, targeted tools designed to identify individuals who may exhibit early signs of dyslexia, allowing for timely diagnostic referral and intervention. However, unlike full diagnostic assessments, which are often resource-intensive and conducted by clinical professionals, screening instruments function as an initial gatekeeping mechanism to detect at-risk learners in educational settings [17,41]. Furthermore, effective dyslexia screening tools must demonstrate both psychometric robustness and practical relevance. This includes content that reflects the core cognitive markers of dyslexia, such as phonological processing deficits, working memory limitations, slow naming speed, and orthographic confusion [6], while remaining accessible to non-specialist users such as teachers. Moreover, face validity is a critical aspect of screening design, as it determines whether items appear, on the surface, to be relevant, understandable, and appropriate to both experts and target respondents [21,29].

Recent trends in dyslexia screening emphasize the need for tools that are developmentally appropriate beyond the early years, particularly for late-identified or late-emerging dyslexia cases in secondary schools [42,43]. Thus, screening instruments must be validated statistically, and through user-centric methods such as face validity evaluation involving both expert panels and student feedback. Additionally, such validation ensures that the instrument captures the lived realities of diverse learners and aligns with inclusive education priorities under SDG 4 [12].

### Dyslexia framework

The development of the dyslexia screening instrument in this study is conceptually grounded in Morton and Frith’s causal model of developmental disorders, which proposes three interrelated levels of explanation: biological, cognitive, and behavioral [44]. According to this framework, dyslexia is not attributable to a single deficit. However, it emerges from interactions across these levels, with the cognitive level serving as the most direct pathway through which difficulties in reading and related skills are expressed. The development of the dyslexia screening instrument was conceptually grounded in Morton and Frith’s causal model of developmental disorders, which emphasizes biological, cognitive, and behavioral levels of explanation. In the present study, particular attention was given to the cognitive level, as this level most directly reflects the learning processes associated with dyslexia in educational contexts. Therefore, although the broader screening framework includes multiple domains, the analysis presented in this paper focuses specifically on items representing cognitive manifestations, such as phonological awareness, working memory, and reading fluency.

The findings indicate that items representing cognitive manifestations of dyslexia were generally perceived as clear and comprehensible by experts and student respondents. This supports the linguistic and conceptual appropriateness of the cognitive-domain items at the preliminary face validity stage. However, further psychometric testing is required before conclusions can be drawn regarding the instrument’s ability to identify students at risk of dyslexia [6,45]. Within this scope, the findings suggest that Morton and Frith’s cognitive-level framework provides a relevant theoretical basis for guiding item development related to dyslexia-associated learning processes in secondary school contexts.

Moreover, situating the instrument within this theoretical framework underscores its relevance beyond item-level clarity by linking early-stage instrument development to broader educational equity concerns. Within the scope of face validity, the findings suggest that theory-driven item development can help ensure that cognitive-domain items are linguistically clear, conceptually aligned, and accessible to intended users. This is consistent with current calls to integrate cognitive-level insights into educational policy and practice to reduce systemic barriers in dyslexia identification [46,47]. In this way, the present study provides preliminary face validity evidence for cognitive-domain items and contributes to the early development of contextually appropriate assessment resources that may support inclusive education goals aligned with SDG 4 [12], pending further psychometric validation.

## Methodology

This study employed an instrument validation research design using a survey among experts and students as test takers, with a specific focus on face validity assessment. Face validity, a crucial psychometric property, measures the degree to which a screening instrument appears appropriate and relevant to its intended users [22]. Furthermore, given the role of early dyslexia detection in ensuring effective interventions, this study validates the FVI of a newly adapted dyslexia screening instrument. The validation process involved a panel of experts who assessed the instrument’s clarity, relevance, and comprehensibility, ensuring its alignment with real-world application in educational and clinical settings [37].

### Ethical considerations

Ethical considerations were observed throughout the face validity assessment process. The study involved expert review for instrument validation purposes only and did not include intervention, experimental manipulation, or collection of sensitive personal information. Ethical approval for data collection involving school-based participants was obtained from the Educational Policy Planning and Research Division, Ministry of Education Malaysia (Approval No: KPM.600-3/2/3-eras (23518), dated 16 Sept 2024), prior to participant recruitment and data collection. Data collection was conducted between September and December 2024.

Institutional ethical clearance was subsequently granted by the UKM Research Ethics Secretariat (Ref No: JEP-2025-416), covering the broader PhD research project. All procedures were conducted in accordance with approved protocols and relevant ethical guidelines. Written informed consent was obtained from all participants prior to data collection. For participants under the age of 18, written consent was additionally obtained from their parents or legal guardians. Participation was voluntary, and participants were informed that their responses would remain confidential and used solely for research purposes.

### Sampling and panels

Prior to the face validity evaluation, the instruments were initially developed in English and underwent a forward-backward translation process to ensure linguistic and contextual equivalence in the Malay version. Hence, to enhance the accuracy and cultural relevance of the translated items, the instrument was reviewed by two certified Language experts, recognized for their expertise in Malay language standardization. It is important to note that the two language experts involved in the translation review were not part of the FVI evaluation panel. Their involvement ensured that the translation maintained semantic equivalence, preventing potential misinterpretations that could affect the validity of the screening tool [22]. This preliminary linguistic validation ensured that the items were semantically accurate, accessible, and appropriate for the target population. Moreover, their inclusion aligns with the principle that face validity refers to the degree to which the assessed items on an instrument accurately correspond to the intended constructs and objectives of the study [48]. Additionally, the data were collected via email over one month.

Subsequently, the verified version was distributed to a panel of ten relevant experts, comprising secondary and primary school teachers. Each had more than ten years of teaching experience [48] in the Malaysian education system and prior involvement in curriculum delivery to students with diverse learning needs. Following the linguistic validation, these expert practitioners were purposively selected based on their pedagogical expertise with at least five years of professional experience and familiarity with the learning profiles of pupils in mainstream and special education settings to refine the instrument further. Notably, the quality of the raters is important, including experience, training experience, and teaching experience [49]. Their primary role was to evaluate the clarity, appropriateness, and comprehensibility of the instrument, particularly in terms of language use and sentence structure. Specifically, each expert was provided with a validation form and rated the instrument items using a 7-point Likert scale, allowing for a quantitative assessment of face validity [21].

However, for the purposes of computing the FVI, the original 7-point Likert responses were recoded into a 4-point format in accordance with methodological recommendations for FVI analysis [20]. Responses representing higher levels of agreement were collapsed to reflect clarity and appropriateness of the items. Specifically, ratings of 1–3 were recoded as 1 (not clear/not appropriate), 4 as 2 (somewhat clear), 5 as 3 (clear), and 6–7 as 4 (very clear/very appropriate). Following established FVI procedures, only ratings of 3 and 4 were considered indicators of agreement when calculating the Item-Level Face Validity Index (I-FVI). This recoding approach ensures alignment with commonly recommended thresholds for face validity evaluation while preserving the interpretability of respondents’ judgments. This expert review process ensured that the instrument was accessible and easily understood by both educators and students, reducing the risk of ambiguity or misinterpretation. Moreover, the heterogeneity of the expert panel allows for a comprehensive evaluation of the instrument’s applicability across different contexts, supporting its usability across diverse linguistic and cultural backgrounds, a key factor in SDG 4 [50]. Table 1 below presents the details of the respective experts.

**Table 1:** Details of experts.

No	Panel Experts	Institution	Expertise	Teaching Experience
**1**	R1	Secondary School	Special Education	10 years
**2**	R2	Secondary School	Special Education	18 years
**3**	R3	Secondary School	Special Education	10 years
**4**	R4	Primary School	Malay Language	13 years
**5**	R5	Secondary School	Malay Language	25 years
**6**	R6	Primary School	Malay Language	10 years
**7**	R7	Secondary School	Malay Language	10 years
**8**	R8	Primary School	Special Education	10 years
**9**	R9	Primary School	Special Education	15 years
**10**	R10	Secondary School	Special Education	25 years

The student panel consisted of ten secondary school students representing the target population of the screening instrument. The students were aged 14 years old and included 4 males and 6 females. The participants were selected to reflect the characteristics of the intended respondents in terms of educational level and language proficiency. Basic demographic information of the participants, including age range, gender distribution, and school level, was recorded to ensure that the feedback reflected the perspectives of the target user group. In face validity studies, a small panel of respondents is generally considered sufficient because the objective is to evaluate item clarity and interpretability rather than to perform statistical generalization. Previous methodological guidelines recommend panels of approximately 5–10 respondents for preliminary face validity assessment [21]. The instrument was subsequently evaluated by ten secondary school students representing the intended end-user group to complement expert-based face validity assessment. The primary purpose of this process was to gather insights directly from test takers regarding the clarity, appropriateness, and interpretability of the item wording, structure, and instructions. Notably, this step is critical in face validation, as it ensures the instrument resonates linguistically and cognitively with its actual users and minimizes the risk of misinterpretation or response bias [23]. Furthermore, the students were instructed to complete the instrument and identify any unclear words or phrases. This was done by a face-to-face approach. [51] mentioned that the face-to-face method is highly effective in improving response rates, while online surveys offer greater efficiency in terms of cost and time. Their feedback was gathered using a 7-point Likert scale to indicate whether they understood each item correctly. Nevertheless, to facilitate the calculation of the FVI, the original scale was transformed into a 4-point Likert format in accordance with widely accepted psychometric conventions. These ratings were used to compute the Item-Level Face Validity Index (I-FVI) and further evaluated through the Scale-Level Face Validity Index by Average Agreement (S-FVI/AVE) and Scale-Level Face Validity Index by Universal Agreement (S-FVI/UA). Moreover, feedback from the students also provided valuable qualitative insights, allowing researchers to refine wording and structure in cases where ambiguity or confusion was observed. This step was crucial in assessing whether the instrument was appropriately tailored to the cognitive and linguistic capabilities of Malaysian students, ensuring that the screening tool effectively serves its intended purpose [25,52]. Consequently, this multi-step validation process enhances the face validity of the dyslexia screening instrument by incorporating expert evaluations and direct feedback from test takers.

Involving test takers in the face validity process reinforces the functional relevance of the instrument and aligns with recommended best practices in scale development, where the cognitive processing and interpretive accuracy of the target population must be prioritized [21,30]. Notably, this approach is critical in educational research involving adolescents, who may vary widely in language proficiency and reading strategies. An informed consent letter was provided to test takers, outlining the objective and procedure of the study while also ensuring participant privacy and data confidentiality.

### Instrument

In particular, the early dyslexia screening instrument used in this study was adapted from two established tools: the Dyslexia Checklist [53] and the Dyslexia Screening for Secondary School Students [54]. The adaptation process was carefully undertaken to ensure the instrument aligns with the linguistic, cultural, and educational context of Malaysia [55,56]. Malaysia is a multicultural and multilingual nation, where numerous languages and dialects are spoken. However, the Malay Language holds primary significance as both the national and official language, functioning as a lingua franca that facilitates communication across diverse ethnic groups [52]. As dyslexia manifests differently across languages and writing systems [13], it was essential to modify the instrument to reflect the phonological and orthographic characteristics of the Malay language. The instrument consisted of 28 items designed to assess cognitive constructs associated with dyslexia, encompassing literacy, numeracy, and general cognitive functioning domains. In this study, these domains are conceptualized as observable manifestations of underlying cognitive processes involved in dyslexia rather than as separate diagnostic constructs. Therefore, the present paper focuses on the cognitive aspects of the screening instrument, particularly items that reflect cognitive difficulties related to reading, working memory, sequential processing, and language-based learning. These items were adapted from an early dyslexia screening instrument specifically developed to identify students exhibiting dyslexic tendencies. Moreover, a seven Likert scale was employed to enhance clarity, ease of administration, and respondent accuracy [29]. The full list of items included in the adapted dyslexia screening instrument is provided as Supplementary Material (S1 File) to enhance transparency and reproducibility.

#### Analysis of data.

All questionnaires returned by the expert panel and student respondents were complete. Therefore, no missing data were identified, and all responses were included in the analysis. Both I-FVI and S-FVI were computed using a structured quantitative approach to determine the face validity of the instrument. This procedure enables researchers to assess the extent to which the items appear clear, appropriate, and understandable to the intended respondents, based on subjective judgment from experts or test takers [21,23].

#### Item level face validity index (I-FVI).

For each item, the I-FVI was recoded as the proportion either ‘3’ (clear/appropriate) or ‘4’ (very clear/very appropriate) on a 4-point Likert scale. The formula is I-FVI = the number of raters in agreement on the item divided by the total number of raters. An I-FVI value of ≥ 0.80 is generally considered acceptable for establishing adequate face validity of individual items [31,57].

#### Scale level face validity index (S-FVI).

Two methods were employed to assess the overall validity of the instrument at the scale level:

1. S-FVI/Average (S-FVI/AVE): This is computed by taking the average of all I-FVI values across items within the same scale. It was calculated by the formula S-FVI/AVE = sum of I-FVI scores divided by the total number of items.
2. S-FVI/Universal Agreement (S-FVI/UA): This index calculates the proportion of items that achieved Universal Agreement (UA), defined as all experts rating the item as 3 or 4. The formula is S-FVI/UA = the number of raters in agreement on an item divided by the number of items.

A value of S-FVI/AVE ≥ 0.90 and S-FVI/UA ≥ 0.80 is commonly recommended to indicate strong scale-level face validity [21,57,58]. Prior to computation, expert panelists’ responses and test takers were recoded into binary values, where scores of 3 and 4 were assigned a value of ‘1’ (indicating agreement that the item is clear and appropriate), and scores of 1 or 2 were coded as ‘0’ (indicating disagreement). This binary recoding facilitates standardized FVI analysis and improves interpretability [31]. No items were removed, and modifications were required for two items (Item 9 and 25), confirming the items’ clarity and comprehensibility.

## Results

The FVI was assessed among expert panelists and test takers to evaluate the clarity, relevance, and comprehensibility of the dyslexia screening instrument. The overall results indicate that the FVI for expert panelists was 0.96, whereas the FVI for test takers was 0.92. Therefore, these findings suggest that the instrument is perceived as clear and understandable by both groups, with expert panelists rating it slightly higher in terms of clarity and ease of understanding. Tables 2 and 3 below illustrate the FVI of experts and test takers, respectively.

**Table 2:** The face validity index of experts.

Item	R1	R2	R3	R4	R5	R6	R7	R8	R9	R10		Experts in Agreement N = 10 experts	I-FVI	UA
1	1	1	1	1	1	1	1	1	1	1		10	1.00	1
2	1	1	1	1	1	1	1	1	1	1		10	1.00	1
3	1	1	1	1	1	1	1	1	1	1		10	1.00	1
4	1	1	1	1	1	1	1	1	1	1		10	1.00	1
5	1	1	1	1	1	1	1	1	1	1		10	1.00	1
6	1	1	1	1	1	1	1	1	1	1		10	1.00	1
7	1	1	1	1	1	1	1	1	1	1		10	1.00	1
8	1	1	1	1	1	1	1	1	1	1		10	1.00	1
9	1	0	1	0	1	1	1	1	0	1		7	0.70	0
10	1	1	1	1	1	1	1	1	1	1		10	1.00	1
11	1	1	1	1	1	1	1	1	1	1		10	1.00	1
12	1	1	1	1	1	1	1	1	1	1		10	1.00	1
13	1	1	1	1	1	1	1	1	1	1		10	1.00	1
14	1	1	1	1	1	1	1	1	1	1		10	1.00	1
15	1	1	1	1	1	1	1	1	1	1		10	1.00	1
16	0	1	1	1	1	1	1	0	0	1		7	0.70	0
17	1	1	1	1	1	1	1	1	1	1		10	1.00	1
18	1	1	1	1	1	1	1	1	1	1		10	1.00	1
19	1	1	1	1	1	1	1	1	1	1		10	1.00	1
20	1	1	1	1	1	1	1	1	1	1		10	1.00	1
21	1	1	1	1	1	1	1	1	1	1		10	1.00	1
22	1	1	1	1	1	1	1	1	1	1		10	1.00	1
23	1	1	1	1	1	1	1	1	1	1		10	1.00	1
24	1	1	1	1	1	1	1	1	1	1		10	1.00	1
25	1	1	1	1	1	1	0	1	1	0		8	0.80	0
26	1	1	1	1	1	1	1	1	1	1		10	1.00	1
27	1	1	1	1	1	1	1	1	1	1		10	1.00	1
28	1	1	1	1	1	0	1	1	0	1		8	0.80	0
**S-FVI-AVE**													**0.96**	
**Proportion**	**0.96**	**0.96**	**1.00**	**0.96**	**1.00**	**0.96**	**0.96**	**0.96**	**0.89**	**0.96**		**S-FVI/UA**		**0.86**
**Average proportion of items judged clarity & comprehension across the 10 raters**											**0.96**			

**Table 3:** The face validity index of test takers.

Item	R1	R2	R3	R4	R5	R6	R7	R8	R9	R10		Experts in Agreement N = 10 Test Takers	I-FVI	UA
**1**	1	1	1	1	1	1	1	1	1	1		10	1	1
**2**	1	1	1	1	1	1	1	1	1	1		10	1	1
**3**	1	1	1	1	1	1	1	1	1	1		10	1	1
**4**	1	1	1	1	1	1	1	1	1	1		10	1	1
**5**	1	1	1	1	1	1	1	1	1	1		10	1	1
**6**	1	1	0	1	1	1	1	1	0	0		7	0.70	0
**7**	1	1	1	1	0	1	1	1	0	0		7	0.70	0
**8**	1	1	1	1	1	1	1	1	1	1		10	1	1
**9**	1	1	0	0	0	0	1	0	0	0		3	0.30	0
**10**	1	1	1	1	1	1	1	1	1	1		10	1	1
**11**	1	1	1	1	1	1	1	1	1	1		10	1	1
**12**	1	1	1	1	1	1	1	1	1	1		10	1	1
**13**	1	1	1	1	1	1	1	1	1	1		10	1	1
**14**	1	1	1	1	1	1	1	1	1	1		10	1	1
**15**	1	1	1	1	1	1	1	1	1	1		10	1	1
**16**	1	1	1	1	1	1	1	1	1	1		10	1	1
**17**	1	1	1	1	1	1	1	1	1	1		10	1	1
**18**	1	1	1	1	1	1	1	1	1	1		10	1	1
**19**	1	1	1	1	1	1	1	1	1	1		10	1	1
**20**	1	1	1	1	1	1	1	1	1	1		10	1	1
**21**	1	1	0	1	1	1	1	1	0	0		7	0.70	0
**22**	1	1	1	1	1	1	1	1	1	1		10	1	1
**23**	1	1	1	1	1	1	1	1	1	1		10	1	1
**24**	1	1	1	1	1	1	1	1	1	1		10	1	1
**25**	1	1	0	1	1	0	0	0	0	0		4	0.40	0
**26**	1	1	1	1	1	1	1	1	1	1		10	1	1
**27**	1	1	1	1	1	1	1	1	1	1		10	1	1
**28**	1	1	1	1	1	1	1	1	1	1		10	1.00	1
**S-FVI-AVE**													**0.92**	
**Proportion**	**1.00**	**1.00**	**0.85**	**0.96**	**0.93**	**0.93**	**0.96**	**0.93**	**0.82**	**0.82**		**S-FVI/UA**		**0.82**
**Average proportion of items judged clarity & comprehension across the 10 raters**											**0.92**			

Across the 28 items, most achieved an I-FVI value of 1.00, indicating unanimous agreement among all ten experts that the items were both clear and appropriate. The data revealed that 24 out of 28 items (85.7%) achieved perfect agreement with I-FVI = 1.00, reflecting unanimous consensus among all raters. Hence, these items indicated optimal face clarity and relevance. However, four items (Items 9, 16, 25, and 28) did not reach full consensus, each scoring an I-FVI of 0.70 or 0.80, and therefore did not meet the UA criterion. Although FVI values ≥ 0.80 are generally recommended, items with FVI values between 0.70 and 0.79 may still be retained during initial development phases, if they are theoretically justified, planned for refinement, and particularly when the number of panelists is 10 or more [59]. Nevertheless, while these items were not eliminated, since they exceeded the minimum threshold of I-FVI ≥ 0.70 [21,30], they were flagged for improvement in terms of semantic precision, sentence structure, and contextual alignment with the literacy profiles of secondary school students. These refinements are crucial in preserving item representativeness without compromising construct integrity.

At the scale level, the S-FVI/AVE for the cognitive construct was 0.96, well above the recommended cutoff of 0.90, indicating consistent clarity across items [58]. Conversely, the S-FVI/UA value was 0.86, confirming that most items were unanimously rated as clear and appropriate by all ten experts. Based on Table 3, the overall results indicated that most items were clearly understood by students, with 23 out of 28 items (82.1%) achieving I-FVI = 1.00, reflecting full agreement among all ten raters. The S-FVI/AVE was calculated at 0.92, exceeding the minimum threshold of 0.90 typically recommended for scale-level clarity [23,58]. Moreover, the S-FVI/UA was 0.82, indicating that most items received unanimous agreement on clarity and comprehensibility.

However, a small number of items, specifically Items 6, 7, 9, 21, and 25, did not achieve full consensus. Most notably, Item 9 recorded the lowest I-FVI (0.30), and Item 25 (I-FVI = 0.40) also fell substantially below the acceptable range. To better understand the low agreement scores, qualitative feedback from student respondents was examined. Students indicated that the wording of Item 9 and Item 25 may have contributed to difficulties in interpretation. Item 9 contains multiple mathematical concepts within a single statement, including combined operations and the use of positive and negative numbers, which may have increased cognitive complexity for respondents. Similarly, Item 25 includes a contextual example (“buying chicken at a roast chicken shop”) that may not be equally familiar to all students, potentially affecting comprehension of the intended construct related to sequential instruction.

The validation logic underlying the retention decision was that low student I-FVI values were treated as evidence of response-process problems, particularly in clarity and comprehensibility, rather than as evidence that the intended construct domains were theoretically irrelevant. Therefore, Items 9 and 25 were not interpreted as having demonstrated adequate face validity in their original form. Instead, they were provisionally retained in the item pool to preserve construct coverage, but only after being flagged for substantial linguistic refinement and subsequent re-evaluation.

In response to the student feedback, both items were refined by simplifying sentence structure, clarifying the subject of the statement, reducing cognitive complexity, and replacing context-specific examples with more general scenarios appropriate for secondary school students. Thus, rather than eliminating these items outright, they were provisionally retained for theoretical completeness and revised to improve accessibility and interpretability. The revised items will require further evaluation through subsequent cognitive interviewing, pilot testing, and psychometric validation before any inference regarding their practical use can be made.

To enhance transparency in the item refinement process, the original wording of the problematic items, the issues identified through student feedback, and the revisions undertaken are presented in Table 4. Although Items 9 and 25 fell below the recommended student I-FVI threshold, they were not treated as face-valid in their original form. Rather, they were provisionally retained because they represented theoretically relevant construct domains, while the low I-FVI values informed targeted linguistic revision and indicated the need for re-evaluation in subsequent validation phases.

**Table 4:** Problematic items and revisions following student feedback.

Item	Original wording	Issue identified	Revised wording
Item 9	I can solve problems that combine different operations and concepts, such as adding and subtracting positive and negative numbers.	Sentence contained multiple mathematical concepts that increased cognitive complexity for students.	I can solve mathematical problems involving different operations, such as adding and subtracting numbers.
Item 25	Can give instructions to others in the correct order. For example, how to buy chicken at a roast chicken shop.	Context-specific example and unclear subject in sentence structure.	I can explain steps or instructions to others in the correct order.

The overall results indicate that the dyslexia screening instrument demonstrates acceptable clarity and comprehensibility from the perspectives of both experts and students. The I-FVI, S-FVI/AVE, and S-FVI/UA values indicate high agreement that most items were clear and understandable, supporting face validity at the preliminary stage of instrument development. Furthermore, these findings provide preliminary evidence of item clarity and user acceptability, supporting the need for subsequent psychometric validation studies to evaluate the instrument’s reliability, construct validity, and screening accuracy. However, it is important to emphasize that the present study focused exclusively on the face validity phase of instrument development. The instrument has not yet been tested using large-scale samples to evaluate its diagnostic accuracy, sensitivity, or specificity in identifying students at risk of dyslexia. Future studies should therefore involve larger and more diverse student populations to examine reliability, construct validity, and screening accuracy before any practical use is considered. A high FVI score (≥0.80) is considered essential in psychometric evaluations, as it enhances the likelihood of stakeholder acceptance and real-world applicability [40].

Improving face validity may enhance item clarity and user acceptability, which are necessary preliminary conditions before further validation and practical implementation are considered [60].

## Discussion

The current study evaluated the face validity of a dyslexia screening tool through a dual-phase approach: expert panel review and test-taker appraisal. This method provides both a top-down theoretical confirmation and a bottom-up perspective of instrument usability, offering a comprehensive validation framework [23,61].

The findings from the expert panel, comprising ten experienced teachers, revealed a high degree of agreement on item clarity and appropriateness. Of the 28 items evaluated, 24 (85.7%) achieved perfect consensus (I-FVI = 1.00), while the remaining four items still met the minimum acceptance threshold (I-FVI ≥ 0.70). Moreover, the S-FVI further confirmed the instrument’s robustness, with S-FVI/AVE = 0.96 and S-FVI/UA = 0.86, exceeding the commonly accepted benchmarks of 0.90 and 0.80, respectively [21,58]. These results indicate that the items are perceived as clear, appropriate, and understandable.

Concurrently, the test-taker-based analysis, involving ten secondary school students, yielded complementary results. A total of 23 items (82.1%) were rated with I-FVI = 1.00, while five items recorded lower agreement, with Item 9 (I-FVI = 0.30) and Item 25 (I-FVI = 0.40) falling notably below acceptable clarity. However, despite these outliers, the S-FVI/AVE remained high at 0.92, and S-FVI/UA at 0.82, indicating that most items were well comprehended by the target population. These findings suggest that most items were perceived as clear and cognitively accessible to students, which is an important preliminary consideration in the development of instruments related to learning difficulties such as dyslexia [41,62]. This finding is consistent with recent review evidence indicating substantial variability in the dyslexia screening tools used in school settings and limited consensus regarding the most effective screening approach [20].

Moreover, the convergence of expert and student perspectives highlights the instrument’s ecological validity and pragmatic usability. On the other hand, items that received high ratings from experts but lower ratings from students (Items 6, 9, and 25) suggest potential mismatches between pedagogical assumptions and actual student comprehension. Hence, these discrepancies underscore the importance of iterative revision, particularly in refining wording, contextual relevance, and linguistic simplicity to ensure that the instrument functions effectively in diverse educational contexts [31].

Notably, this dual-validation approach aligns with Messick’s unified theory of validity, which posits that test validation must account for both the content and substantive aspects of score interpretation [61]. It also reflects best practices in contemporary instrument development, which advocate for stakeholder involvement, including end users as a core component of scale refinement [23,30].

In the context of educational equity, this process holds relevance to SDG 4, which emphasizes inclusive and equitable quality education for all. Therefore, by examining whether the cognitive-domain items were perceived as clear and accessible by both experts and students, this study contributes to efforts aimed at closing diagnostic gaps, particularly for older students who may otherwise remain unidentified within the system [12]. Consequently, such early-stage validation work may contribute to the development of instruments that can later be evaluated for their usefulness in educational support pathways.

The findings have practical implications for the early-stage development of school-based dyslexia screening instruments. Specifically, the results suggest that most items were perceived as clear, understandable, and acceptable by both educators and students. However, because the present study was limited to face validity, these findings should not be interpreted as evidence of screening accuracy, diagnostic utility or practical effectiveness. Rather, the instrument may proceed to subsequent validation phases, including reliability testing, construct validation, and evaluation of screening accuracy, before it can be considered for use in school-based identification or referral processes.

### Limitation and future directions

This study was intentionally designed as an initial phase of instrument validation, with a specific emphasis on face validity to establish item clarity, relevance, and user acceptability among secondary school students. Methodological decisions, including the size of the expert panel and test-taker group, were guided by established recommendations for Face Validity Index (FVI) analysis, which prioritize expert judgment and depth of evaluation rather than large sample sizes at the early stages of instrument development. However, the relatively small number of student raters involved in the face validity assessment may introduce some degree of statistical instability in the estimation of I-FVI values. Therefore, the findings should be interpreted as preliminary evidence of item clarity and comprehensibility rather than definitive psychometric validation of the instrument. The focus on face validity reflects a deliberate and theoretically grounded approach, recognizing that user acceptance and interpretability are foundational prerequisites before advancing to more complex psychometric evaluations. While advanced analyses such as construct validity, reliability testing, and item-level modelling were beyond the scope of the present study, these procedures are planned as subsequent phases to further strengthen the instrument’s measurement properties. Additionally, the development and evaluation of the instrument within the Malaysian secondary school context ensured linguistic and cultural appropriateness, providing a controlled foundation for future cross-cultural validation and broader application.

## Conclusions

This study provides empirical evidence supporting the face validity of the cognitive-domain items of an adapted dyslexia screening instrument for secondary school students. Through the integration of expert evaluations and test-taker feedback, the findings demonstrate that the instrument exhibits high levels of clarity, relevance, and user acceptability. The I-FVI and S-FVI indices indicate high agreement on item clarity, comprehensibility, and user acceptability among experts and student respondents. These findings support face validity as a preliminary validation step, but they do not establish the instrument’s screening accuracy, diagnostic utility, reliability, or construct validity. Further studies involving larger and more diverse samples are required before the instrument can be considered for practical implementation in educational settings.

Importantly, the findings highlight the value of incorporating face validity as a foundational component in instrument development, particularly for screening tools intended for practical educational use. By prioritizing item comprehensibility and stakeholder acceptance, the study addresses a frequently overlooked aspect of psychometric validation that is critical for successful implementation in real-world contexts. This is especially relevant in secondary schools, where dyslexia often remain unidentified until academic demands intensify.

Although the present study focused specifically on face validity, it represents a deliberate and necessary first phase in the validation process. The instrument represents an early-stage item pool that may be further evaluated for its potential role in school-based identification and referral processes. In this respect, the study contributes to the broader agenda of Sustainable Development Goal 4 [12], which emphasizes inclusive and equitable quality education by supporting early identification and reducing barriers to learning for students with specific learning difficulties. Future research will extend this work through advanced psychometric analyses to further strengthen the instrument’s measurement properties and applicability across diverse educational contexts.

Overall, this study advances evidence-informed approaches to the development of dyslexia screening instruments and contributes to efforts aimed at promoting inclusive education. However, it is important to emphasize that face validity represents only an initial stage of instrument development. Further studies involving larger samples are necessary to examine the instrument’s reliability, construct validity, and diagnostic accuracy before any screening-related interpretation or practical implementation in educational settings is considered.

## Supporting information

S1 File Early dyslexia screening questionnaire cognitive. (PDF)
