# Supplementary material for: Face validity of a dyslexia screening instrument for secondary students
Source: PLoS One. 2026 Jul 20;21(7):e0353781. doi: 10.1371/journal.pone.0353781 (PMC13384271; doi:10.1371/journal.pone.0353781)
Supplement: S1 File — (PDF) [file pone.0353781.s001.pdf]

QUESTIONNAIRE ON LEARNING PROCESSES AND PSYCHOSOCIAL FACTORS AMONG SECONDARY SCHOOL STUDENTS

SECTION A: RESPONDENT DEMOGRAPHICS

Instructions: Please respond by marking (/) in the space provided.

i) PERSONAL INFORMATION

**Name**	
**Gender**	
Male	
Female	
**Age (Please state: ....)**	
**Race**	
Malay	
Chinese	
Indian	
Others (Please state:________________________________)	

SECTION B: COGNITIVE

Instructions: Please choose **ONE** answer and mark (/) either YES or NO.

No	Literacy	1	2	3	4	5	6	7
1	I often confuse words that sound similar when reading, like "*sayang*", "*layang*", "*bayang*", and "*dayang*".							
2	When I read a passage, I have trouble remembering the sentence from the previous paragraph.							
3	I often mix up some words while reading a paragraph. For example, I might read "dengan" as "lengan".							
4	When reading, I sometimes skip syllables. For example, "*bebola*" becomes "*bola*", and "*makanan*" becomes "*makan*".							
5	I get confused with similar-looking numbers when reading, like 273,689.							
6	I find it hard to tell apart words with similar sounds when reading. For example, "*besi*" becomes "*basi*".							
7	I have difficulty pronouncing words with prefixes and suffixes, like "*pengemudian*" or "*pemejalwapan*".							
8	I struggle to spell unfamiliar words, such as "*penyahaktifan*".							
	**Numeracy**	**1**	**2**	**3**	**4**	**5**	**6**	**7**
9	I can solve problems that combine different operations and concepts, such as adding and subtracting positive and negative numbers (e.g. 6 + 8 - 9).							
10	I can do addition and subtraction in my head without writing it down.							
11	I often fail to read measurements on a scale, like volume.							
12	I often fail to read measurements on a scale, like square measurements.							
13	I can solve math problems that involve more than one method.							
14	I understand information presented through charts or graphs.							
15	I cannot keep to the time limit during class activities, such as finishing an exercise within 15 minutes.							
16	I find it hard to solve word problems in math. For example, I take a long time to convert time to the 24-hour system.							

	General Cognitive Functioning	1	2	3	4	5	6	7
17	I find it hard to remember the names of people I just met.							
18	I cannot explain something clearly.							
19	I can state the main ideas from a passage I read.							
20	I can combine words to form complete sentences. For example, turning "*paku batu*", "*paku kayu*", "*abu*", and "*ada*" into "*Abu ada paku kayu dan paku batu*".							
21	I often change the meaning of a sentence without realizing it.							
22	I take a long time to understand a poster when reading it.							
23	I get confused and don't understand voice messages on the phone.							
24	I take a long time to understand a brochure when reading it.							
25	I can give instructions to others in the correct order. For example, how to buy chicken at a roast chicken shop.							
26	I can understand even when someone talks fast.							
27	I can describe my journey to school.							
28	I can complete a picture rearrangement activity within the time set by the teacher.							

Foot note:

Words in Italic in this instrument are intentionally not translated and retained in the Malay language to preserve the original linguistic mechanics relevant to reading-related processes among students at risk of dyslexia in the Malaysian context.

THANK YOU ☺
